# Influence of the Intention to Lean the Body Forward on Kinematics and Kinetics of Sprinting for Active Adults

**DOI:** 10.3390/sports7060133

**Published:** 2019-05-31

**Authors:** Ryu Nagahara, Elaheh Amini, Kelly Cristina Cesco Marcon, Peng-Wen Chen, Jessica Chua, Jens Eiberger, Nathaniel Jonathan Claridad Futalan, Jamie Lye, Marko Milan Pantovic, Michal Starczewski, Kriyot Sudsa-ard, Sri Sumartiningsih, Chien-Yen Wang, Tania Beverly William, Tonnie Kasujja, Tariq Ali Gujar

**Affiliations:** 1 National Institute of Fitness and Sports in Kanoya, Kanoya 891-2393, Japan; 2 Center for Sport and Exercise Science, University of Malaya, Kuala Lumpur 50603, Malaysia; elaheham@yahoo.com; 3 Autonomous Sport Confederation of Guatemala, Guatemala 33102, Guatemala; kel.marcon@gmail.com; 4 Institute of Sports Sciences, University of Taipei in Taiwan, Taipei 11114, Taiwan; a0928438940@gmail.com; 5 Exercise and Sport Studies, Nanyang Technology University, Singapore 639798, Singapore; jessica.chua@swimming.org.sg; 6 Myanmar Football Federation, Yangon 11072, Myanmar; jens-eiberger@web.de; 7 Philippine Sports Commission, Manila 1004, Philippine; n_futalan@yahoo.com; 8 National Youth Sports Institute, Singapore 737913, Singapore; jamie_lye@nysi.org.sg; 9 Faculty of Sport and Physical Education, University of Belgrade, 11000 Belgrade, Serbia; marko.sportscience@gmail.com; 10 Physiology Department, Institute of Sport National Research Institute, 01-982 Warsaw, Poland; michalstarczewski1@gmail.com; 11 Faculty of Allied Health Science, Thammasat University, Phathumthani 12121, Thailand; kriyot.s@allied.tu.ac.th; 12 Sports Science Department, Semarang State University, Semarang 50229, Indonesia; sri.sumartiningsih@gmail.com; 13 Graduate Institute of Sports Coaching Science, Chinese Culture University, Taipei 11114, Taiwan; 14 Institute of Athletic and Coaching Science, National Taiwan Sport University, Taoyuan 33301, Taiwan; cirewcirew@gmail.com; 15 Global Sport Management, Seoul National University, Seoul 08826, Korea; taniabeverly@yahoo.com; 16 Tsukuba International Academy for Sport Studies, University of Tsukuba, Tsukuba 305-8574, Japan; kasujjat@gmail.com; 17 Sports Science Department, Otto Von Guericke University Magdeburg, Magdeburg 39106, Germany; t_gujar@yahoo.com

**Keywords:** manipulation, running, training, technique, instruction

## Abstract

This study investigated the influence of the intention to lean the body forward on spatiotemporal and ground reaction force variables during the acceleration phase of a sprint. Fourteen active adults performed two 50 m sprints (with and without the intention to lean), during which spatiotemporal variables and impulses were obtained using a long force platform system. Effect size (Cohen’s d) was used to examine the differences between the two trials. We found that running speed and net anteroposterior impulse did not change by the intention for all steps. However, step frequency increased in the initial two steps through decreases in support time and flight time by the intention. Moreover, these shorter support and flight times were caused by a decrease in the vertical impulse. The propulsive impulse did not change during the initial part of acceleration phase, but the braking impulse decreased at the first step. This study demonstrates that an intention to lean the body forward leads to a smaller braking impulse and a higher step frequency through shorter support and flight times and a smaller vertical impulse during the initial part of the acceleration phase of a sprint.

## 1. Introduction

Sprinting is an essential ability in many sports and is a basic exercise in a physical education class. In a 100 m race, the finish time is strongly correlated with maximal speed [1], thus achieving a high maximal speed should produce a better performance. Because the maximal speed is a result of preceding acceleration, the determinants of effective acceleration have broadly been investigated [2,3,4,5,6,7,8].

To accelerate to a high maximal speed, sprinters theoretically need to produce a substantial positive net anteroposterior ground reaction force (GRF) during the support phase of each step. However, a greater resultant force is not necessarily accompanied by a greater horizontal propulsive force [9]. Kugler and Janshen [9] found that faster athletes produce a smaller resultant force compared to slower athletes, but the GRF vector of the faster athletes is directed more horizontally than that of slower athletes. That is, the direction of the GRF is more important than its magnitude for effective acceleration. This concept has been confirmed in recent studies [8,10,11,12], and to direct the GRF vector more horizontally is one of the key instructions for improving sprint acceleration performance from a practical perspective.

Although the aforementioned studies provided basic knowledge for improving sprint acceleration performance, there is a lack of knowledge on how athletes can achieve better sprint acceleration performance through an intentional change in running motion or direction of force production. To the best of our knowledge, there is only one study that investigated the immediate effect of an intention on sprint acceleration performance [13]. Bezodis et al. [13] investigated maximal effort 10 m sprinting in three conditions (control, internal focus, and external focus), following different verbal instructions intended to manipulate the force vector direction, using team sports athletes. However, they reported that intentional changes did not improve sprint acceleration performance. This shows the difficulty of improving sprint acceleration performance through instructions within the short term. In contrast to sprinters and team sports athletes, physically active adults who do not train for competitions have greater margins for improving sprinting performance. Thus, a study of these cohorts is more likely to produce a detectable change in sprint acceleration performance and its determinants. Moreover, from a physical education standpoint, the findings from untrained participants could be more useful than those from well-trained athletes.

The aim of the present study was to investigate the influence of the intention to lean the body forward on spatiotemporal and GRF variables during sprint acceleration in active adults. The hypothesis was that, because greater acceleration is accompanied by a forward-leaning posture [5,9,14,15], instructing the athlete to lean the body forward would be effective in improving sprint acceleration performance within a single session. The investigation of the influence of the intention to lean the body forward on spatiotemporal and GRF variables during sprint acceleration was expected to provide useful knowledge for sprinters, coaches, and teachers for the development of technical training regimens to improve sprinting performance.

## 2. Materials and Methods

### 2.1. Participants

Six female and eight male active adults (mean ± SD: age, 30.6 ± 6.0 years; stature, 1.69 ± 0.13 m; body mass, 72.0 ± 16.1 kg) participated in this study. The aim, risks of involvement, and experimental conditions of the study were explained to the participants before the experiment, and written informed consent was obtained from the participants. The experimental procedures were conducted with approval from the research ethics committee of the institute.

### 2.2. Experiments

After a warm-up, the participants who wore their own running shoes performed two maximal effort 50 m sprints from a crouched standing position (pre- and post-trial). Because the participants were not familiarized with a crouched starting position with hands on the ground using blocks, the crouched standing position was adopted for the start. The rest period between the trials was 15 min. The post-trial was performed with the intent of producing a greater propulsive force with the intention to lean the whole body forward. Fifty-two force platforms (1000 Hz) connected to a single computer (TF-90100, TF-3055, Tec Gihan, Uji, Japan) collected GRFs during sprinting across a 50 m distance. The experimenter provided the participants with a signal for the initiation of the sprints.

### 2.3. Data Processing

GRF signals were smoothed using a Butterworth low-pass digital filter at a cut-off frequency of 50 Hz in reference to previous studies [4,6,7]. Spatiotemporal variables at each step were calculated from GRF data. Foot strike and toe-off were determined using a vertical GRF with a threshold of 20 N [4,6,7]. The duration of each step was determined from the foot strike of one leg to the next foot strike of the other leg. Step frequency (SF) was computed as the inverse of step duration. Support time (ST) was defined as the duration from the foot strike to toe-off, and flight time (FT) was defined as the duration from the toe-off to foot strike. The location of the ground contact foot was determined as the center of pressure of GRF on the ground at the middle (0.1 s) of the support phase of each step [4,6,7]. Step length (SL) was calculated as the distance between ground contact foot locations for two serial steps in the anteroposterior direction, and running speed was calculated as the product of SL and SF. To evaluate the sprinting performance for the entire distance, the mean running speed was computed by averaging running speed of all the steps. Propulsive and braking impulses were calculated using time integrations of the positive and negative anteroposterior forces with the trapezoid formula. The net anteroposterior impulse was calculated as the sum of the propulsive and braking impulses. The vertical impulse was obtained using the time integration of the vertical GRF with the trapezoid formula.

To cancel bilateral differences and human cyclic movement variability, we approximated spatiotemporal and GRF variables against the time axis using a fourth-order polynomial, in reference to previous studies [2,3,6,7]. As the average raw running speed for all participants in pre- and post-trials reached the maximal speed at the 20th step, the data until the 20th step were extracted for all participants for approximation, which allowed us to test the differences in variables at each step across all participants. Before the approximation, we added the mean value of the last two steps (i.e., the 19th and 20th steps) for three steps after the 20th step, to eliminate the influence of endpoint error. The duration of the last step was used as the time intervals for these added data. Values from the start phase (from the start signal to the toe-off of the front leg at the start) were eliminated from the approximation to avoid the challenges associated with distinguishing the phases of steps and detecting the initiation of motion, as the start phase was a double support movement and there were large differences in starting styles.

### 2.4. Statistical Analyses

The means and SDs of the approximated spatiotemporal and GRF variables, as well as those of time and distance, were calculated at each step. A simple two-tailed t-test was used to compare the mean running speed between the two trials with a significance level of p < 0.05. Cohen’s d [16] as a measure of effect size (ES) of the difference in variables was used to examine differences in spatiotemporal and GRF variables between the two trials at each step. The smallest worthwhile change was determined as an effect size of 0.2 [17,18].

## 3. Results

**Figure 1:**
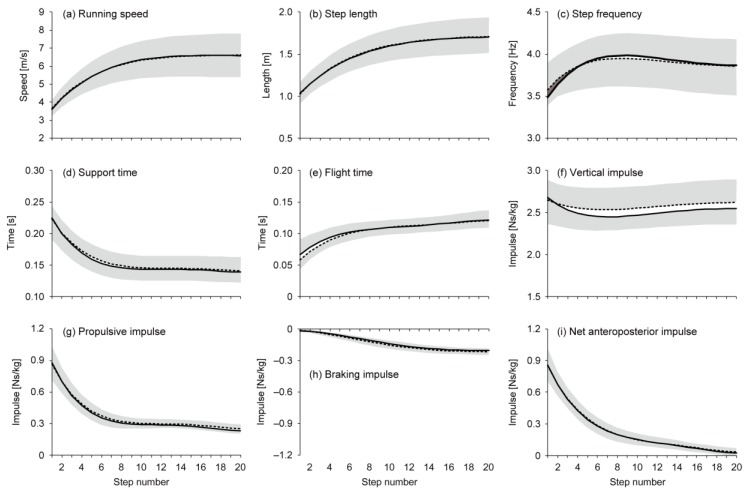
Mean and standard deviation of the approximated (**a**) running speed, (**b**) step length, (**c**) step frequency, (**d**) support time, (**e**) flight time, (**f**) vertical impulse, (**g**) propulsive impulse, (**h**) braking impulse, and (**i**) net anteroposterior impulse for 20 steps. Mean values are plotted at each step. Dotted and solid lines represent the pre- and post-trials, respectively. The grey shaded area represents standard deviation for each of the pre- and post-trials.

**Figure 2:**
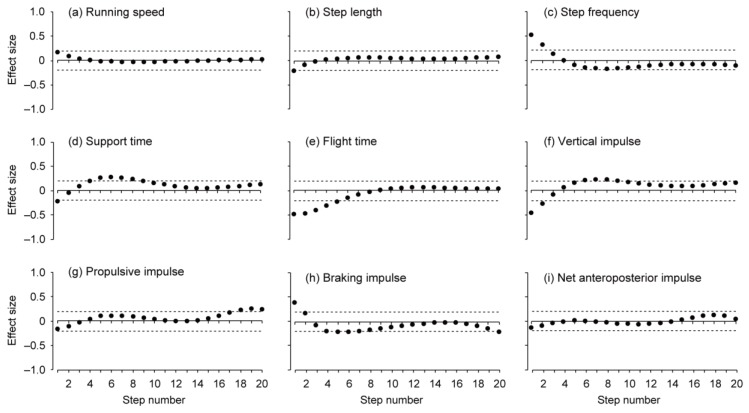
Effect sizes between the pre- and post-trials at each step for (**a**) running speed, (**b**) step length, (**c**) step frequency, (**d**) support time, (**e**) flight time, (**f**) vertical impulse, (**g**) propulsive impulse, (**h**) braking impulse, and (**i**) net anteroposterior impulse. Horizontal dotted lines indicate the smallest worthwhile change (0.2).

There was no significant difference (p = 0.766, ES = 0.01) in the mean running speed for 50 m between the pre- (5.88 ± 0.99 m/s) and post-trials (5.87 ± 0.92 m/s). For eight of the 14 participants, the faster trial occurred in the pre-trial. Figure 1 shows changes in the approximated spatiotemporal and GRF variables for the pre- and post-trials (noting that the steps of the added data are not shown). Time and distance at the 20th step for the post-trial were 5.82 ± 0.46 s and 28.5 ± 3.2 m, respectively. The maximal speeds (6.59 ± 1.21 and 6.61 ± 1.23 m/s in the pre- and post-trials, respectively; hereafter, values of the pre- and post-trials appear in the same order) were reached at the 19th and 20th steps for pre- and post-trials, respectively (Figure 1a). For running speed, there was no difference between the two trials for all steps (Figure 2a). SL for both trials reached maximum values (1.70 ± 0.24 and 1.71 ± 0.20 m) at the 20th step (Figure 1b), and it was shorter for the post-trial at the first step (Figure 2b). SF reached maximum values (3.99 ± 0.23 and 3.94 ± 0.33 Hz) at the eighth and ninth steps in the pre- and post-trials (Figure 1c), and it was higher for the post-trial at the initial two steps (Figure 2c). ST showed minimum values (0.139 ± 0.017 and 0.141 ± 0.021 s) at the 20th step for both trials (Figure 1d), and it was shorter at the first step and longer from the fourth to ninth steps for the post-trial (Figure 2d). FT reached maximum values (0.121 ± 0.016 and 0.121 ± 0.010 s) at the 20th step for both trials (Figure 1e), and it was shorter for the post-trial from the first to fifth steps (Figure 2e). The vertical impulse was smaller at the initial two steps and greater from the sixth to ninth steps in the post-trial than in the pre-trial (Figure 2f). The propulsive impulse was greater during the last three steps in the post-trial than in the pre-trial (Figure 2g). The braking impulse was smaller at the first step and greater at the fifth, sixth, and 20th steps in the post-trial than in the pre-trial (Figure 2h). The net anteroposterior impulse did not show a difference between the two trials (Figure 2i).

## 4. Discussion

This study was the first to investigate the influence of the intention to lean the body forward on sprint acceleration performance within the short term. Using active adults, spatiotemporal and GRF variables were obtained during 50 m sprint acceleration. While there was no change in running speed, some underlying components of sprinting changed, indicating that the intention to lean the body forward could change the locomotor pattern.

In this study, running speed did not change at all steps during sprint acceleration through the intention to lean the body forward. This indicates that the verbal instruction to lean the body forward within a short term (single session) is not effective for improving sprint acceleration performance for active adults. Our finding of no improvement in sprint acceleration performance with verbal instructions agrees with a report by Bezodis et al. [13], who examined the influence of internal focus and external focus intended to manipulate the force vector direction on a 10 m sprint performance using team sports athletes. The findings from the current and previous studies show the difficulty of improving sprint acceleration performance through verbal instructions within a short term. However, even though sprint performance in the study by Bezodis et al. [13] was impaired through the instruction within short-term, sprinting performance in the present study was maintained. Although it is difficult to explain the reason for this discrepancy, the difference in characteristics between the cohorts of the previous study and the current study (team sports athletes in the previous study vs. active adults in this study) is a possible reason.

Although there was no difference in running speed, SL decreased and SF increased during the initial few steps when there was an intention to lean the body forward. Moreover, ST at the first step and FT at the initial five steps decreased, and ST from the fourth to ninth steps increased through the intention. These results demonstrate that the intention to change body posture may result in a change in locomotor pattern without producing a change in running speed. Because SF is calculated as an inverse of step duration and the step duration consists of ST and FT, the decreases in ST and FT probably caused the increase in SF during the initial acceleration section. Moreover, because the vertical impulse decreased at the initial two steps and smaller vertical impulse is accompanied by higher SF [19], it seems that the increase in SF through the decreases in ST and FT resulted from the smaller vertical impulse. Accordingly, the intention to lean the body forward could lead to a higher SF and shorter ST and FT through a smaller vertical impulse during the initial acceleration phase. Hunter et al. [20] found that a smaller relative vertical impulse was accompanied by a smaller backward lean of the whole leg at the foot strike during sprint acceleration around the 16 m mark. Kugler and Janshen [9] found that faster athletes produced a smaller vertical force with a greater forward lean of the whole body during the initial acceleration compared to slower athletes. Thus, it is possible that the intention to lean the body forward in this study resulted in a greater forward lean of the support leg and the whole body, as well as a smaller vertical impulse.

Regarding GRF variables in the anteroposterior direction, net anteroposterior impulse did not change at all steps when there was an intention to lean the body forward. While the braking impulse decreased at the first step, it increased at the fifth and sixth steps. A smaller braking impulse with greater forward leaned leg posture has been found in a previous study [20]. Moreover, an intention to lean the body forward possibly leads to the backward foot strike position, which results in a smaller braking force. Accordingly, it is likely that the intention to lean the body forward would induce a smaller braking impulse during the initial acceleration section. An athlete needs to raise the body to the upright position through the acceleration phase, and trying to keep the forward lean body posture during the initial acceleration section imposes extra needs to raise the body to the upright position during the subsequent section. A greater rise of body posture needs a longer ST and thus causes a greater braking impulse. Thus, it is reasonable that the intention to lean the body forward was accompanied by a greater braking impulse after the initial acceleration section during the sprint acceleration. Although the changes in braking and propulsive impulses did not affect the magnitude of the net anteroposterior impulse, understanding these characteristic changes in GRF variables during sprint acceleration when having the intention to lean the body forward would be beneficial when trying to improve sprinting performance with manipulation of the running motion. Whereas the braking and propulsive impulses increased at the last few steps through the intention to lean the body forward, running speed just before the maximal speed changes slightly and these changes would not influence the entire performance of the sprint acceleration.

Regarding the limitations of this study, data were used until the 20th step, at which point some participants had not reached their maximal speed. However, changes in running speed close to the maximal speed were quite small and; therefore, the range of steps investigated in this study could be considered as the entire acceleration phase. This study used active adults as participants. Thus, there is a possibility that the results might differ when sprinters or team sports athletes are examined. Although we did not randomize the order of trials (with or without the intention) to avoid learning effect and to simulate training or physical education class situation, it is possible that there was an influence of fatigue or extra warm-up. Because this study did not collect running motion data, differences in body posture and leg kinematics could not be discussed. Thus, it would be interesting to examine the changes in running motion in association with the changes in GRF variables in the future. Finally, this study examined the influence of the intention to lean the body forward within a short term, while the influence of the intention to lean the body forward within long-term training is still unknown.

## 5. Conclusions

The current study demonstrates that an intention to lean the body forward leads to consistent sprint acceleration performance during the entire acceleration phase, a shorter step length, and higher step frequency through shorter support and flight times during the initial acceleration section, and longer support time and greater vertical and braking impulses during the subsequent section in sprint acceleration. The findings in this study will be a reference for coaches, athletes, and teachers who try to improve sprint acceleration performance through manipulating sprinting techniques.
